# Screening of Natural Extracts for Inhibitors against Japanese Encephalitis Virus Infection

**DOI:** 10.1128/AAC.02373-19

**Published:** 2020-02-21

**Authors:** Jiao Guo, Xiaoying Jia, Yang Liu, Shaobo Wang, Junyuan Cao, Bo Zhang, Gengfu Xiao, Wei Wang

**Affiliations:** a State Key Laboratory of Virology, Wuhan Institute of Virology, Center for Biosafety Mega-Science, Chinese Academy of Sciences, Wuhan, China; b University of the Chinese Academy of Sciences, Beijing, China

**Keywords:** high-content screening (HCS), Japanese encephalitis virus (JEV), ouabain, digoxin, Na^+^/K^+^-ATPase

## Abstract

The mosquito-borne Japanese encephalitis virus (JEV) causes serious illness worldwide that is associated with high morbidity and mortality. Currently, there are no effective drugs approved for the treatment of JEV infection. Drug-repurposing screening is an alternative approach to discover potential antiviral agents. In this study, high-content screening (HCS) of a natural extracts library was performed, and two hit FDA-approved Na^+^/K^+^-ATPase inhibitors, ouabain and digoxin, were identified as having robust efficiency against JEV infection with the selectivity indexes over 1,000. The results indicated that ouabain and digoxin blocked the JEV infection at the replication stage by targeting the Na^+^/K^+^-ATPase. Furthermore, it was proven that ouabain significantly reduced the morbidity and mortality caused by JEV in a BALB/c mouse model. This work demonstrated that Na^+^/K^+^-ATPase could serve as the target of treatment of JEV infection, and ouabain has the potential to be developed as an effective anti-JEV drug.

## Abstract (precis)

The mosquito-borne Japanese encephalitis virus (JEV) causes serious illness worldwide that is associated with high morbidity and mortality. Currently, there are no effective drugs approved for the treatment of JEV infection. Drug-repurposing screening is an alternative approach to discover potential antiviral agents.

## INTRODUCTION

Japanese encephalitis virus (JEV) is a member of the genus *Flavivirus* in the family *Flaviviridae*, containing a single-stranded, positive-sense RNA genome of approximately 11 kb in length, which encodes three structural proteins, including the capsid (C), membrane (prM and M), and envelope (E), and seven nonstructural proteins (NS1, NS2A, NS2B, NS3, NS4A, NS4B, and NS5) (1).

JEV has been a global health concern since it was first discovered in the late 19th century (2), with an estimated 68,000 clinical cases and 10,000 to 15,000 associated deaths per year (3, 4). JEV infection-related clinical symptoms include fever, headache, vomiting, diarrhea, mental status changes, and signs of meningeal irritation (5); the ratio of survival with psychiatric sequelae or permanent neurological is approximately 44% (6). Although the morbidity and mortality decreased with the use of various vaccines, and several recent researchers have identified a number of compounds with anti-JEV activities, such as *N*-nonyl-deoxynojirimycin (7), dehydroepiandrosterone (8), *N*-methylisatin-beta-thiosemicarbazone derivative (SCH 16) (9), indirubin (10), manidipine (11), chlorpromazine (12), etanercept (13), and minocycline (14), few therapies beyond intensive supportive care to treat patients with JEV are available.

To address the urgent need for anti-JEV therapy, a high-content screen (HCS) assay was performed with a library of 1,034 natural extracts, which contains untapped reservoirs of potent and novel inhibitors and activators of biological pathways, for discovering inhibitors against JEV infection. Two hit FDA-approved compounds, ouabain and digoxin, were identified to inhibit JEV infection both *in vitro* and *in vivo*. The results of this study are expected to contribute to our understanding of the biology of JEV infection and provide novel host targets for the development of anti-JEV drugs.

## RESULTS

### Optimization of HCS assay conditions.

The HCS assay-optimized conditions contain cell density, infective dose, and assay endpoint. The assay conditions for HCS were optimized as follows: cell density of 10,000 cells per 96-well plate, a multiplicity of infection (MOI) of 0.8, and an endpoint of 24 h postinfection. The signal-to-basal (S/B) ratio, coefficient of variation (CV), and Z factor were 2,177, 8.9%, and 0.72, respectively, which demonstrated that the assay was robust and reproductive for the large-scale screening of novel antiviral compounds against JEV infection.

### HCS assay of drug library screening.

**FIG 1:**
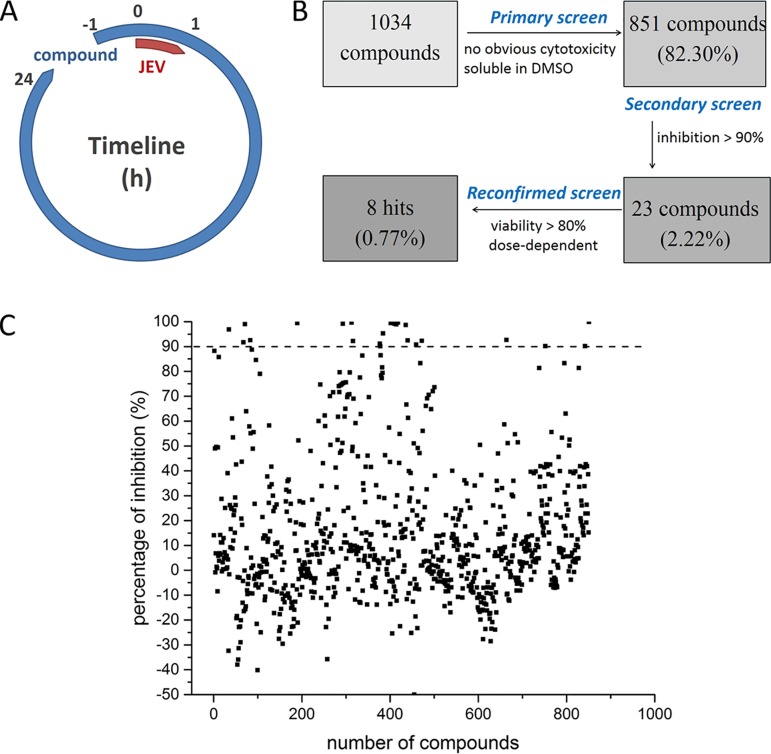
HCS for inhibitors of JEV infection using the natural extracts library. (A) HCS assay timeline. (B) HCS assay flowchart. (C) HCS of a library of 1,034 natural extracts for primary candidates inhibiting JEV infection. Each dot represents the percent inhibition achieved with each compound at a concentration of 50 μM.

**FIG 2:**
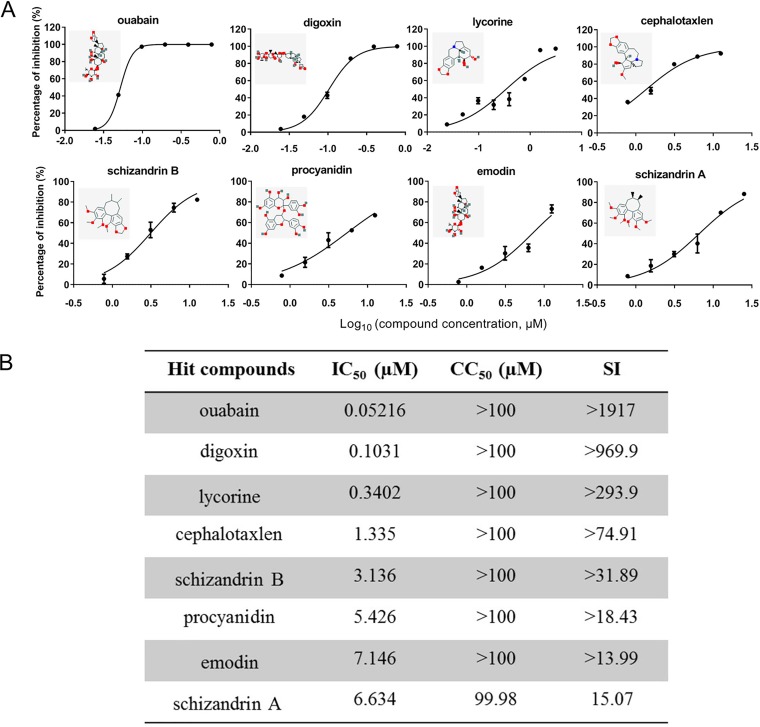
Inhibitory effects of the hit compounds. (A) Dose-response curves of 8 hits for inhibition of JEV infection. (B) IC_50_s, CC_50_s, and SIs of 8 hit compounds selected from the reconfirmation screen. Data are represented as the means ± standard deviations (SDs) from at least two independent experiments.

To reveal novel targets and inhibitors against JEV infection, an immunofluorescence assay (IFA)-based HCS assay was performed by screening a library of 1,034 natural extracts for the anti-JEV activities (Fig. 1A; Table S1). As shown in Fig. 1B and C, 851 compounds were selected for the second screening because of their solubility in dimethyl sulfoxide (DMSO) at room temperature and absence of any obvious cytotoxicity on Vero cells. Compounds (50 μM) that exerted a >90% inhibition against JEV were defined as prime candidates; based on this criterion, 23 compounds (2.22%) were selected. A screening to reconfirm the results was then carried out using these prime candidates over a broader concentration range (3.125 to 50 μM). Eight hit compounds (0.77%) were selected based on their concentration-dependent inhibitory effects and a cell viability of >80% (Fig. 2A and B). Among the 8 compounds, ouabain and digoxin were subjected to further investigation because of their high selective indexes (SI, which is defined as 50% cytotoxic concentration [CC_50_]/50% inhibitory concentration [IC_50_]) and similar mechanisms (namely, they are cardiac glycoside compounds and act as inhibitors of Na^+^/K^+^-ATPase) of >1,917 and >969.9, respectively.

### Antiviral effects of ouabain and digoxin in different cell lines and viruses.

**FIG 3:**
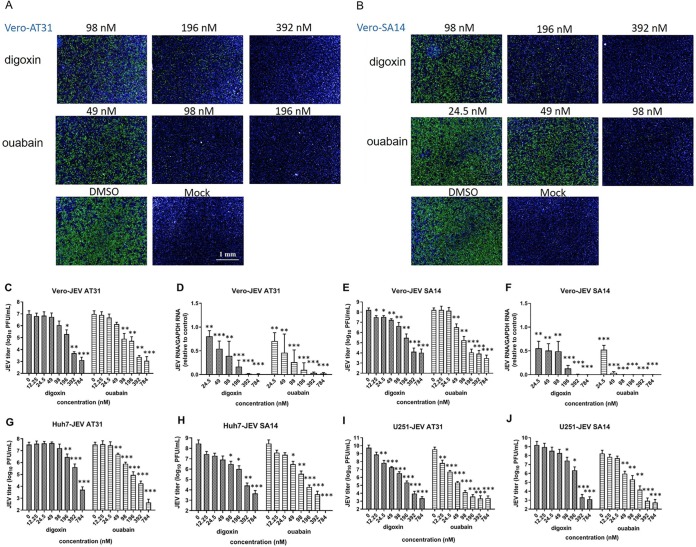
Validation of the antiviral effects of ouabain and digoxin in different cell lines and virus strains. The antiviral effects of digoxin and ouabain against two JEV strains (AT31 and SA14) in Vero cells. (A and B) Immunofluorescence staining assay (IFA) images showing the viral protein (green) and nuclei (blue) are displayed. (C and E) The Vero cell culture supernatants were subjected to plaque assay. (D and F) The Vero cell lysates were assessed by qRT-PCR. (G to J) The antiviral effects of digoxin and ouabain against two JEV strains in Huh-7 cells and U251 cells, respectively. The cell culture supernatants were collected to perform the plaque assay. Data are represented as the means ± SDs from at least three independent experiments. The scale bar indicates 1 mm. ***, *P* < 0.001; **, *P* < 0.01; *, *P* < 0.05.

For AT31 infection of Vero cells, digoxin (392 nM) and ouabain (196 nM) displayed inhibition rates >90%; the ability to block the virus reduced with a decrease in concentration (Fig. 3A). Similarly, SA14 reproduction could be greatly inhibited when the concentration of digoxin and ouabain reached 392 nM and 98 nM, respectively, Simultaneously, the fluorescence signal increased with diminishing drug concentrations (Fig. 3B).

Under the same conditions of infection, ouabain and digoxin robustly inhibited AT31 and SA14 virus production in a plaque assay, with a reduction of approximately 4 to 6 log units at the highest concentration (Fig. 3C and E). A sharp decrease in JEV RNA levels was also detected with a quantitative reverse transcription PCR (qRT-PCR) assay. In particular, the attenuated RNA levels of the SA14 strain in the high-dose-, middle-dose-, and low-dose-treated groups were all above 40%, indicating a strong inhibition of viral replication (Fig. 3D and F).

Further, the inhibitory effect of two compounds on AT31 and SA14 infection was also evaluated in Huh-7 cells and U251 cells. In line with JEV-infected Vero cells, plaque assays were performed to detect the viral titer, and a dose-dependent anti-JEV effect was similarly observed in the two cell lines (Fig. 3G to J).

### Ouabain and digoxin inhibit JEV infection during viral RNA synthesis.

**FIG 4:**
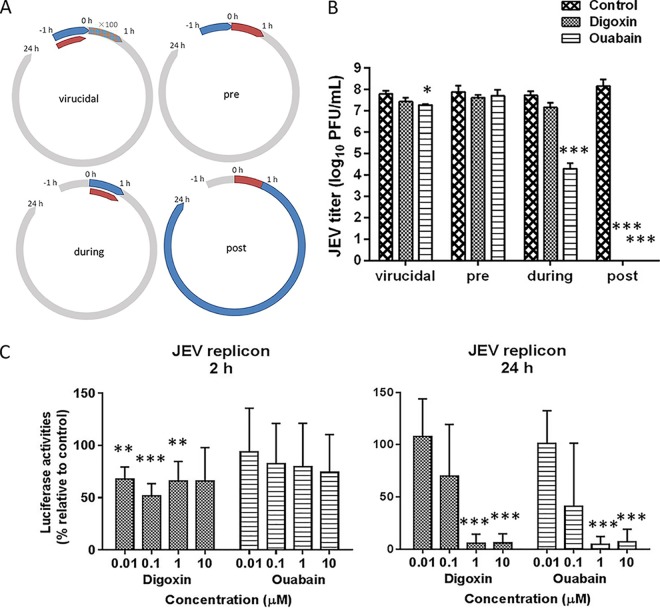
Time-of-addition experiment and JEV reporter replicon assay of the antiviral activities of ouabain and digoxin. (A) Schematic illustration of the time-of-addition experiment. (B) The inhibitory effects of digoxin and ouabain with the indicated concentration in each group, determined by plaque assay. (C) Huh-7 cells transfected with the JEV replicon were treated with digoxin and ouabain, respectively, and luciferase activities were determined as indicated. Data are represented as the means ± SDs from at least three independent experiments. ***, *P* < 0.001; **, *P* < 0.01; *, *P* < 0.05.

To determine the cellular process that these drugs target, a time-of-addition experiment was performed (Fig. 4A). As shown in Fig. 4B, neither digoxin nor ouabain showed suppression of JEV in virucidal treatments or pretreatments. However, ouabain showed a robust inhibition of JEV during treatment. Most importantly, when added posttreatment, both ouabain and digoxin exerted full inhibitory effects on JEV infection, suggesting that viral replication was the main stage at which these drugs showed inhibitory activity.

To confirm this hypothesis, Huh-7 cells electroporated with the JEV replicon were treated with the indicated concentration of ouabain and digoxin; we revealed the appreciable reduction of luciferase signal at 24 h postelectroporation (Fig. 4C). Our results indicated that both drugs inhibited JEV RNA synthesis in a dose-dependent manner, while neither drug inhibited the initial translation of replicon RNA, confirming that these drugs inhibit JEV infection at the replication stage.

### JEV inhibition with ouabain and digoxin occurs via Na^+^/K^+^-ATPase.

**FIG 5:**
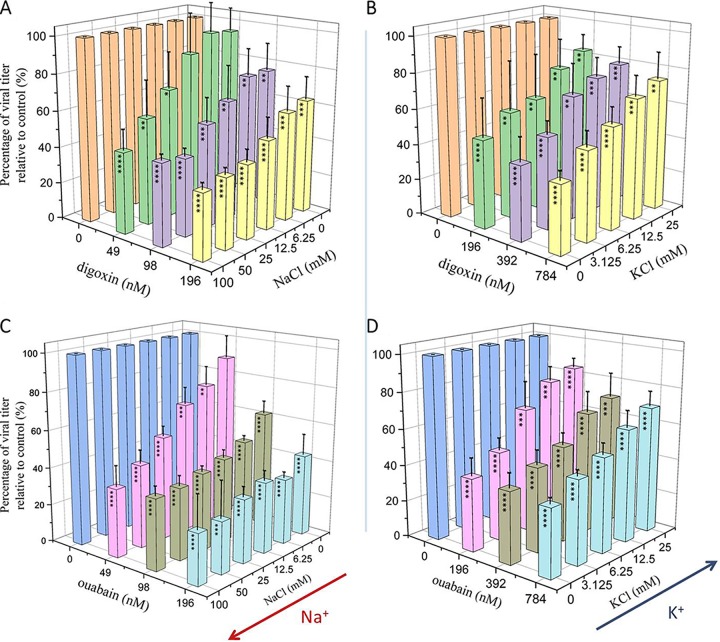
Inhibition of JEV with digoxin and ouabain occurs via Na^+^/K^+^-ATPase. Vero cells infected with JEV AT31 were treated with increasing concentrations of NaCl (A and C) and KCl (B and D) at 1 h preinfection. At 24 h postinfection, cell supernatants were collected for the plaque assay. Data are represented as the means ± SDs from at least three independent experiments. ****, *P* < 0.0001; ***, *P* < 0.001; **, *P* < 0.01; *, *P* < 0.05.

Na^+^/K^+^-ATPase is the only target of cardiac glycosides that has been found to date. To confirm whether JEV inhibition occurs via blockade of Na^+^/K^+^-ATPase, the infected cells were treated with DMSO and two compounds in the presence of increasing NaCl and KCl, and two compounds inhibited JEV in a dose-responsive manner. Furthermore, at the same concentration of digoxin, JEV inhibition reduced with the decrease of NaCl (Fig. 5A) but with an increase of KCl (Fig. 5B). Under the same experimental conditions, the results of ouabain action were consistent with digoxin (Fig. 5C and D), which indicated that the JEV-inhibiting effect of both compounds is positively correlated with extracellular NaCl but inversely correlated with KCl. These results confirm that both ouabain and digoxin exert the antiviral effect by targeting the Na^+^/K^+^-ATPase.

### Ouabain protects against JEV infection-induced lethality *in vivo*.

**FIG 6:**
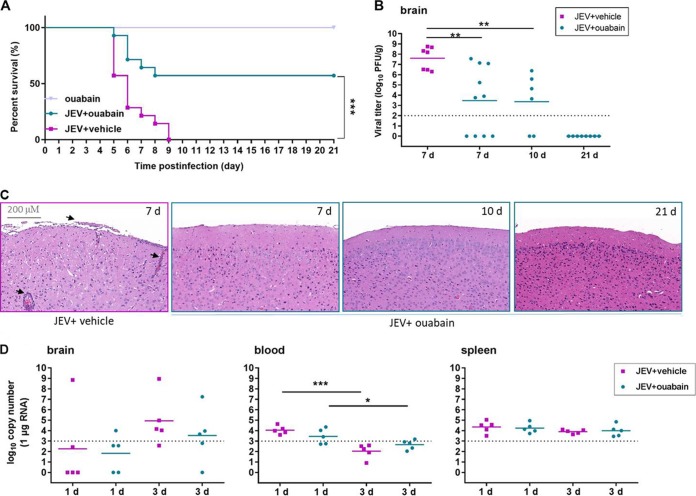
Ouabain protected mice from JEV infection. BALB/c mice were infected with 1 × 10^6^ PFU of JEV together with ouabain or vehicle by intraperitoneal route. (A) Survival of mice in each group was monitored for 21 days after inoculation of JEV by intraperitoneal injection. Data are shown as Kaplan-Meier survival curves (*n* = 14 to 15 for each group). (B) The viral loads in mouse brains were measured by plaque assay on days 7, 10, and 21. (C) Ouabain treatment alleviated the histopathological changes in mice caused by JEV infection. Arrows indicate the histopathological changes such as meningitis, perivascular cuffing, and glial nodules. (D) The viral loads in brain, blood, and spleen were measured by qRT-PCR on days 1 and 3, respectively. Dashed lines indicate limit of detection. The scale bar indicates 200 μM. ***, *P* < 0.001; **, *P* < 0.01; *, *P* < 0.05.

As ouabain exhibited a stronger inhibitory activity on JEV infections, we further examined the protective effect of ouabain on JEV-induced lethality by using the BALB/c mouse. As anticipated, mice in the JEV-infected, vehicle-treated group started to show symptoms including limb paralysis, restriction of movement, piloerection, body stiffening, and whole-body tremor at day 5 postinfection. Within 9 days of infection, all mice in the JEV-infected group succumbed to the infection. Ouabain treatment following JEV infection increased the survival rate to nearly 60% (8 out of 14 animals survived) (Fig. 6A). Mice treated with ouabain alone showed little abnormal behavior. These results suggest that ouabain provides effective protection against JEV-induced mortality.

To further relate these protective effects to viral load and histopathological changes in mouse brains, the viral titer was determined and mouse brain sections were collected and assayed at days 7, 10, and 21 postinfection. The results indicated that, during the progression of the disease, ouabain treatment significantly reduced viral load in infected mice compared to untreated infected mice. Notably, no plaques formed in the ouabain-treated group on day 21 postinfection (Fig. 6B). Similarly, apparent damage in the brain, including meningitis, perivascular cuffing, and vacuolar degeneration, was observed in the JEV-infected and vehicle-treated group on day 7 postinfection, while ouabain treatment remarkably alleviated these phenomena (Fig. 6C). These results indicate that alleviation of histopathological changes is accompanied by a reduction in the viral load as well as a reduction in mortality rate, further confirming the curative effects of ouabain on viral encephalitis.

The viral loads of brain, blood, and spleen were also evaluated at the early stage of infection. As shown in Fig. 6D, on day 1 postinfection, viral titers in the brain were almost undetectable, and ouabain exhibited little effect on viral titers in the brain at the preclinical stage. Accordingly, ouabain had little effect on decreasing viral titers in the blood and spleen at the earlier time points, which was mostly due to that the detectable viral genome copy numbers were too low to be comparable. Notably, titers in the blood decreased sharply from day 1 to day 3 postinfection, which was in line with the characteristic viremia caused by JEV. These results indicate that ouabain could alleviate histopathological changes and reduce viral loads in the brain, thus protecting mice against JEV-induced lethality and confirming the curative effects of ouabain on viral encephalitis.

## DISCUSSION

JEV continues to spread in Asia and other parts of the world, causing severe clinical manifestations such as permanent neurological or psychiatric sequelae. Currently, there is no safe and effective treatment for JEV infection. Recently, Wang et al. performed a library screen of 1,018 FDA-approved compounds and reported that manidipine, a calcium blocker using in the treatment of hypertension, inhibited JEV infection *in vitro* and *in vivo* (11). Similarly, Fang et al. tested 1,280 FDA-approved drugs and found that FGIN-1-27, an anxiolytic drug that targets the peripheral benzodiazepine receptor, reduced the JEV infection *in vitro* (15). Drug screening and repurposing has become a very useful approach for identifying antiviral drugs, as it explores novel molecular targets to study virus pathogenesis.

To address the urgent need for anti-JEV therapy, we presented a library of natural extracts to check for the ability to inhibit JEV infection. Our high-content screening assay design could identify compounds that inhibit JEV viral entry, translation, and RNA synthesis. In this study, eight hit compounds with SI indexes greater than 10 were found to exert inhibitory effects on JEV. Among these eight compounds, some were previously reported to possess a wide spectrum of pharmacological effects, including antiviral activity. Moreover, some compounds, such as lycorine, emodin, and procyanidin, have been proven to be effective in inhibiting flavivirus or HCV infections via different mechanisms (16–20). These results demonstrate that our HCS assay was effective and credible.

The top two compounds, FDA-approved Na^+^/K^+^-ATPase inhibitors ouabain and digoxin, are cardiac glycosides with similar chemical structures and have been used for the treatment of cardiac arrhythmias and hypotension for more than 200 years. Recently, ouabain and digoxin have been proven to inhibit different kinds of viruses, including enveloped viruses such as coronaviruses, nonenveloped viruses such as reoviruses, DNA viruses such as human cytomegalovirus, positive-sense RNA viruses such as chikungunya virus, and negative-sense RNA viruses such as lymphocytic choriomeningitis virus (LCMV) (21–25). Notably, we have tried to select drug-resistant variants by serial passaging of JEV using increasing concentrations of digoxin and ouabain, respectively. However, no adaptive mutant was found after 25 passages with either drug. This result suggested that both drugs might exert the antiviral effects by targeting the cellular protein other than the viral protein, making the barrier to resistance more difficult to overcome.

Cardiac glycoside acts via inhibiting the sodium-potassium ion pump, leading to changes in the intracellular concentration of sodium, potassium, and calcium, which have been shown to play essential roles in many cellular biosynthetic signaling and vesicular sorting pathways (26). In this study, ouabain exhibited therapeutic effects on JEV infection in an adult mouse model by decreasing viral loads and alleviating pathological injuries in the brain, which significantly improved the survival rate of JEV-infected mice. We proposed two mechanisms that may contribute to the antiviral effect*s*. First, ouabain may block JEV infection by inducing the cellular stress response. Ouabain treatment in mammalian cells causes the interaction between the inositol 1,4,5-trisphosphate (IP3) receptor and Na^+^/K^+^-ATPase to induce calcium oscillations and further activate calcium-dependent transcription factors, such as the NF-κB and activator protein (27). Some evidence suggested that cardiac glycosides mediate inflammatory processes via the activation of the phosphoinositide 3-kinase/Akt pathway and the Src/mitogen-activated protein kinase pathway (28, 29). These signaling pathways also cause the activation of NF-κB. Many of the genes expressed were elicited by activation of NF-κB, including innate immune response, growth, and differentiation, which stimulate an antiviral state that might block JEV infection in the end (30). Second, JEV could replicate in the central nervous system (CNS), and infections result in the breakdown of the blood-brain barrier (BBB) along with an influx of inflammatory cells; the breakdown of BBB caused by JEV infection may have allowed BBB-nonpermissive ouabain to enter the brain, bind the murine ATPase α2 and α3 isoforms (31), and inhibit viral replication in neurons.

Together, these findings provide valuable information that may be used in future clinical trials examining the effects of ouabain on JEV infection. Moreover, our results indicate that ATPase is a promising pharmacological target in JEV infection.

## MATERIALS AND METHODS

### Ethics statements and mice.

All animal experimental procedures were carried out according to ethical guidelines and were approved by the Animal Care Committee of the Wuhan Institute of Virology (permit number, WIVA25201705).

### Cells and viruses.

BHK-21, Vero, and Huh-7 cells were maintained in Dulbecco modified Eagle medium (DMEM) (HyClone, Logan, UT, USA) supplement with 10% fetal bovine serum (FBS) (Gibco, Grand Island, NY, USA). U251 cells were maintained in minimum essential medium (MEM) (HyClone) supplement with 10% FBS. JEV AT31 and SA14 strains were generated by using the infectious clones of pMWJEAT-AT31 (kindly provided by T. Wakita, Tokyo Metropolitan Institute for Neuroscience) and pACYC-JEV-SA14 (GenBank accession no. U14163) as previously described (32), respectively. The infectious clone plasmids were linearized, subjected to *in vitro* transcription, and electroporated into BHK-21 cells. Three days later, the supernatant was collected and stored at −80°C in aliquots (33, 34). The virus stocks were propagated and titrated by a plaque assay in BHK-21 cells.

### Optimization of HCS assay conditions.

The cell density, infective dose, and assay endpoint were optimized for the HCS assay. Different densities (2,000 to 10,000 cells per well) of Vero cells were infected at MOI values ranging from 0.2 to 1. Cell viability was detected at different times (24 to 72 h) after JEV inoculation. The appropriate cell density, infective dose, and assay endpoint for the HCS assay were selected by comparing the signal-to-basal ratio (S/B), the coefficient of variation (CV), and *z* values under different conditions as previously described (11); 10 μM manidipine and 0.5% DMSO were used as positive and negative controls, respectively.

### HCS assay of drug library screening.

A library of 1,034 compounds from natural extracts was purchased from Weikeqi Biotech (Sichuan, China). Compounds were stored as 10 mM stock solutions in DMSO at –80°C until use. As shown in Fig. 1A, Vero cells were dissociated and seeded at a density of 1 × 10^4^ cells per well in 96-well plates. After overnight incubation, cell monolayers were treated in duplicate with the compounds at a final concentration of 50 μM for 1 h and infected with the JEV strain AT31 at an MOI of 0.8. After 1 h infection, the supernatant was replaced with a fresh medium, and compounds were added to the same wells. After an additional 23 h of incubation, infection was stopped by rinsing each well once with phosphate-buffered saline (PBS) and fixing the cells with 4% paraformaldehyde. Cells were permeabilized using PBS with 0.2% Triton X-100 for 15 min and blocked with 5% FBS (Gibco), followed by treatment with the primary antibody anti-JEV prM (full-length prM was expressed in Escherichia coli BL21 cells using pET30a expression vectors, and purified protein was injected into rabbits to prepare polyclonal antibodies) overnight at 4°C. After six rinses with PBS, the cells were stained with DyLight 488-labeled antibody to rabbit IgG (KPL, Gaithersburg, MD, USA). Nuclei were counterstained by DAPI (4′,6-diamidino-2-phenylindole) (Sigma-Aldrich, USA). Nine fields per well were imaged on an Operetta high-content imaging system (PerkinElmer), and the percentages of infected and DAPI-positive cells were calculated using the associated Harmony 3.5 software.

### Antiviral effects of ouabain and digoxin with different cell lines and virus strains.

Vero, Huh-7, and U251cells in 96-well plates were infected with JEV strain AT-31 or SA14 at an MOI of 0.8 at different concentrations for 24 h. The IFA assay was performed as described above, while qRT-PCR and the plaque assay were carried out as previously reported (35).

### Time-of-addition experiment.

The time-of-addition experiment was performed as previously described (11) (Fig. 4A). Vero cells were infected with JEV strain AT31 for 1 h (0 to 1 h). Ouabain (10 μM) and digoxin (10 μM) were incubated with the cells for 1 h or 23 h according to the following: preinfection (−1 to 0 h), during infection (0 to 1 h), and postinfection (1 to 24 h). To exclude a possible direct inactivating effect of the drugs, viruses were incubated with each drug (1 μM) at 37°C for 1 h, and the mixtures were diluted 100-fold before infection of Vero cells. Virus titers were determined by plaque assay 24 h later.

### JEV replicon assay.

To ensure the effectiveness of the two hit drugs in JEV replication, SA14 (GenBank accession no. U14163) replicon cDNA clone in which the structural genes were replaced with the *Renilla* luciferase (*Rluc*) gene was employed to quantitatively evaluate the inhibitory effects (32). *In vitro* transcripts were synthesized from linearized JEV replicon using a T7 mMessage mMachine kit (Albion, Austin, TX) according to the manufacturer’s instructions. Huh-7 cells were electroporated with *in vitro* transcripts in 800 μl of Gene Pulser Electroporation buffer (Bio-Rad) for electroporation cuvettes with a 4-mm electrode gap and a Gene Pulser II (Bio-Rad) unit at settings of 250 V and 950 μF, pulsing 1 time. After electroporation, the cells were plated in DMEM supplemented with 10% FBS, and compounds were added to the medium when specified. At the indicated times postelectroporation, the cells were harvested, and luciferase activity was measured using the Rluc assay system (Promega, Madison, WI).

### Addition of sodium and potassium assay.

Vero cells seeded in 96-well plates were treated with DMSO or either compound diluted in a medium supplemented with NaCl (at a concentration of 0, 6.25, 12.5, 25, 50, or 100 mM) and KCl (at a concentration of 0, 3.125, 6.25, 12.5, or 25 mM) for 1 h, respectively. Then JEV AT31 with an MOI of 0.8 was added in each well. After 1 h, the cell supernatants were removed and treated with the indicated concentration of each compound in an ion-supplemented medium for an additional 23 h, and the virus titers were determined by plaque assay using BHK-21 cells. Inhibition rates are calculated as the percentage of infected cells normalized to DMSO-treated cells for at least triplicate repeat experiments.

### Antiviral efficacy of ouabain in BALB/c mice.

Adult male BALB/c mice (4 weeks) were randomly divided into three groups: the JEV-infected and vehicle (0.75% DMSO in normal saline)-treated group, the JEV-infected and ouabain-treated group, and the ouabain-treated group. For infection, mice were infected intraperitoneally (i.p.) with a dose of 1 × 10^6^ PFU JEV (AT31). The infected mice were i.p. administered with ouabain at 3 mg/kg of body weight or a vehicle control every day for 21 consecutive days. Mice were sacrificed at day 1, 3, 7, 10, or 21, depending on the experimental design. Blood, spleen, and brain samples were collected for plaque assay, qRT-PCR, and histopathology investigation (Vectra ss; PerkinElmer).

The viral burden was determined by plaque assay. The samples were weighed and homogenized in 300 μl of PBS. The homogenates were clarified by centrifugation (2,000 × *g* at 4°C) for 15 min and then diluted serially prior to infection of BHK-21 cells. The viral titer was adjusted for sample volume and tissue weight to calculate the titer as PFU/g.

Tissue samples and blood from JEV-infected mice were extracted with the RNAprep pure tissue kit (Tiangen; catalog no. DP431) and RNAprep pure blood kit (Tiangen; catalog no. DP433), respectively. JEV RNA levels were determined by one-step qRT-PCR on ABI Real-Time PCR systems using standard cycling conditions. The viral burden was calculated on a standard curve produced using serial 10-fold dilutions of plasmids carrying the infectious clone.

For histopathology investigation, brain tissues were collected from the mice and fixed in 4% paraformaldehyde; the hematoxylin-eosin staining protocol was followed for tissue staining.

### Statistical analysis.

Student's *t* test and log rank tests were used to evaluate the statistical significance of differences. A value of *P* < 0.05 was considered statistically significant.

## Supplementary Material

Supplemental file 1
